# Predominance of* Acinetobacter pseudolwoffii* among *Acinetobacter* species in domestic animals in the Czech Republic

**DOI:** 10.17221/65/2023-VETMED

**Published:** 2023-11-29

**Authors:** Vladimir Sladecek, David Senk, Petr Stolar, Jaroslav Bzdil, Ondrej Holy

**Affiliations:** ^1^Ptacy s.r.o, Valasska Bystrice, Czech Republic; ^2^Department of Infectious Diseases and Preventive Medicine, Veterinary Research Institute, Brno, Czech Republic; ^3^Science and Research Centre, Faculty of Health Sciences, Palacky University Olomouc, Olomouc, Czech Republic

**Keywords:** organ, prevalence, pathogenicity, species, susceptibility, veterinary

## Abstract

The aim of this study was to map the spectrum of microorganisms belonging to the genus *Acinetobacter* in domestic animals with a specific focus on the prevalence of *Acinetobacter pseudolwoffii.* Additionally, the susceptibility of isolates to antimicrobial agents was determined. In the period from January 1, 2014, to August 31, 2015, a total of 9 544 samples originating from gross lesions and pathological processes of animals exhibiting clinical symptoms of the disease were examined across 41 districts in the Czech Republic. The examinations were carried out using culture methods involving meat-peptone blood agar and Endo agar under aerobic conditions at a temperature of 37 ± 1 °C for 18–24 hours. Isolates were confirmed using molecular phenotypic method MALDI–TOF MS with the MBT Compass Library Revision L 2020 covering 3 239 species/entries (9 607 MSP) from Bruker Daltonics company. Out of the 108 isolates (prevalence 1.13%), 14 species of *Acinetobacter* spp. were identified, with 5 isolates remaining unclassified as species. *A. pseudolwoffii* was the predominant species isolated in 25 cases (prevalence 0.26%). Antimicrobial susceptibility was determined for 12 antimicrobials by the disc diffusion method, with *A. pseudolwoffii* isolates exhibiting the lowest susceptibility to ceftazidime (32%) and co-trimoxazole (60%).

*Acinetobacter* spp. comprising 76 species (Euzeby 2023) are ubiquitous microorganisms found in soil, water, and clinical environments. In humans and animals, they are primarily commensals colonising the skin and gut of patients and hospital staff, and contaminating hospital equipment (Bergogne-Berezin et al. 2008). Some species can cause local (e.g. wound infections) and systemic diseases (Nemec 1999; Almasaudi 2018). In human medicine, the *Acinetobacter calcoaceticus*/*baumannii* complex (ACB complex) is of major epidemiological importance (Nemec et al. 2015). Less common species, such as *Acinetobacter parvus* (Nemec et al. 2003), *Acinetobacter guillouiae* (Nemec et al. 2010), and *Acinetobacter modestus* (Nemec et al. 2016) have been isolated from various human cultures. *Acinetobacter* spp. are commonly isolated from animals, including birds and fish (Almasaudi 2018) and can lead to various diseases, sometimes with fatal consequences (Francey et al. 2000). In the Czech Republic *Acinetobacter pittii*, *Acinetobacter calcoaceticus*, *Acinetobacter towneri*, *Acinetobacter johnsonii*, *Acinetobacter lwoffii* and other unspecified species of the genus *Acinetobacter* have been isolated from pathological processes and gross lesions in horses with a prevalence ranging from 0.2% to 2.2% (Bzdil et al. 2018). The isolation of *Acinetobacter pseudolwoffii* strains was first described by Nemec et al. (2019) in ruminants, horses, guinea pigs, humans, and environmental samples. The increase in resistance in *Acinetobacter* spp. and the emergence of multi-drug resistant strains in human medicine is worrying (Kurcik-Trajkovska 2009). Multi-drug resistance was also recorded in animals (Jokisalo et al. 2010). A detailed description of the susceptibility of *Acinetobacter* spp. isolates obtained from gross lesions and processes in horses in the Czech Republic is provided by Bzdil et al. (2018). In dogs and cats, strains of *A.* *baumannii* resistant even to carbapenems were found (Gentilini et al. 2018). The objective of this study was to map the spectrum of species in the genus *Acinetobacter* in a wide range of samples collected from animals with clinical signs of disease and to describe the susceptibility of the isolates to antimicrobial agents.

## MATERIAL AND METHODS

Over the 20-month period from January 1, 2014, to August 31, 2015, a total of 9 544 clinical samples were collected from pathological processes and lesions in animals displaying disease symptoms originating from farms in 41 districts of the Czech Republic. Veterinarians at 13 clinics and veterinary hospitals and 18 private veterinarians were instructed to collect the samples from a wide variety of animals, including dogs, cats, cattle, sheep, goats, pigs, equids, i.e. horses and donkeys, waterfowl, fowl, exotic birds, rabbits, guinea pigs, mice, rats (both kept as pets), hamsters, snakes, turtles, tortoises, lizards and even bees (bee brood). However, no clinical samples from fish were included in this examination.

The collected samples included swabs or irrigations from eyes and ears, swabs and scrapings from the skin, swabs from the respiratory system, sputum and bronchioalveolar lavages, swabs from the digestive system, faeces, urine, swabs from the mucous membranes of the urogenital system, mammary gland secretions, milk, blood, swabs from the heart and blood vessels, as well as swabs and punctures from the musculoskeletal, lymphatic and nervous systems. Table 1 presents a summary of the quantities and types of clinical materials collected. Liquid and slurry materials were collected in sterile plastic containers with a volume of 60–200 ml, or in sterile plastic tubes with a 10 ml capacity and a screw cap (Dispolab Ltd., Brno, Czech Republic). Swabs were taken using the Transbak swab system containing Amies agar with activated carbon (Dispolab Ltd., Brno, Czech Republic). After collection, the samples were kept at a temperature of +4 °C to +6 °C and transported to the laboratory within 24 h, where they were immediately processed. A culture examination was performed on meat-peptone blood agar (MPBA) and Endo agar (EA), both from Trios s.r.o. (Prague, Czech Republic). The inoculated plates were incubated aerobically at a temperature of 37 ± 1 °C for 18–24 hours. Suspect strains were isolated and confirmed by MALDI–TOF MS using a Microflex LT System spectrometer (Bruker Daltonics GmbH, Bremen, Germany) and evaluated with the MBT Compass Library Revision L 2020 covering 3 239 species/entries (9 607 MSP) (Bruker Daltonics GmbH, Bremen, Germany). Identification scores (ID) within the range of 2.300 to 3.000 were evaluated as highly probable for species identification, 2.000 to 2.299 as secure genus identification and probable species identification, 1.700 to 1.999 as probable genus identification, and values ≤ 1.699 as unreliable identification. The identification of *A.* *baumannii* was confirmed by the multiplex PCR method through the detection of *bla*_OXA-51_ and *bla*_OXA-51-like_ genes encoding natural carbapenemases, which are specific to this species (Turton et al. 2006). Pure cultures were tested for susceptibility to antimicrobial substances using the disc diffusion method on Mueller-Hinton agar (MH) (Trios s.r.o., Prague, Czech Republic) and discs (Oxoid Ltd., Basingstoke, UK). Of the antimicrobial substances, imipenem (10 μg), meropenem (10 μg), tobramycin (10 μg), netilmicin (30 μg), ofloxacin (5 μg), amikacin (30 μg), doxycycline (30 μg), ampicillin/sulbactam (20 μg), gentamicin (10 μg), ceftazidime (30 μg), co-trimoxazole (25 μg) and piperacillin (100 μg) were tested. Tests were assessed after 16–18 h of incubation at 35 ± 1 °C. Interpretation of values was performed according to CLSI (2020) standards. Reference values for *Pseudomonas aeruginosa* were used to assess susceptibility to netilmicin and ofloxacin. The quality of the media and discs was validated by reference strains of *Escherichia coli* (ATCC 25922), *P.* *aeruginosa* (ATCC27853), and *Staphylococcus aureus* (ATCC 25923).

**Table 1:** Number of veterinary clinical samples examined in the period from January 1, 2014 to August 31, 2015

Animal	Domestic carnivores	Domestic pigs	Domestic solipeds	Domestic birds	Domestic rodents	Domestic reptiles	Domestic insects (bee)	Total No. of samples
Organ (system)								
Eye	179	2	11	2	12	0	0	234
Ear	597	0	0	0	1	0	0	599
Skin	486	0	35	4	12	5	0	578
Respiratory	208	33	85	24	64	5	0	544
Digestive	705	82	16	262	79	28	0	1 419
Urogenital	120	0	2	9	0	0	212	344
Mammary gland	6	0	0	0	0	0	0	5 756
Circulation	0	2	0	17	0	0	0	22
Musculoskeletal	18	1	4	13	1	0	0	39
Lymphatic	1	0	2	2	0	0	0	7
Nervous	0	2	0	0	0	0	0	2
Total No. of samples	2 320	122	155	333	169	38	212	9 544

## RESULTS AND DISCUSSION

Out of 9 544 clinical samples, 108 *Acinetobacter* isolates were obtained during the observed period (prevalence 1.13%). A total of 14 *Acinetobacter* species were identified: *Acinetobacter baumannii*, *Acinetobacter calcoaceticus*, *Acinetobacter gandensis*, *Acinetobacter guillouiae*, *Acinetobacter indicus*, *Acinetobacter johnsonii*, *Acinetobacter lwoffii*, *Acinetobacter modestus*, *Acinetobacter parvus*,* Acinetobacter pittii*, *Acinetobacter pseudolwoffii*, *Acinetobacter radioresistens*, *Acinetobacter schindleri*, and *Acinetobacter ursingii*. Five isolates were not classified as species. The species *A.* *gandensis* and *A.* *modestus* have not yet been reported in animals or in products of animal origin in the literature. In our study, no *Acinetobacter* spp. isolates were found in sick pigs during the observed period. This could be attributed to the smaller number of samples collected from these animals. In addition, samples from the respiratory system mainly originated from the lungs, where the probability of finding *Acinetobacter* spp. tends to be lower. Samples from the digestive tract were frequently overgrown with saprophytic microflora making the isolation of individual *Acinetobacter* spp. problematic.

In terms of organs and organ systems, *Acineto-bacter* spp. isolates were not found in the samples from the musculoskeletal, lymphatic, and nervous systems of the animals. Of the species we observed, *A.* *pseudolwoffii* was predominant (*n* = 25) with a prevalence of 0.26%. The prevalence of this species cannot be compared with other data because no similar studies exist currently. The second and third positions were held by the species *A.* *lwoffii* (*n* = 21) and *A.* *pittii* (*n* = 19) with prevalences of 0.22 and 0.2%, respectively. Despite the relatively high number of detected *A.* *lwoffii* and *A.* *pittii* isolates in our study, their prevalence is comparatively lower or similar to that reported in other studies (Bzdil et al. 2018; Samkange et al. 2022). The finding of *A.* *pseudolwoffii* isolates was first described by Nemec et al. (2019) in nasal swabs from a cow, a calf, a goat, and a horse, in a rectal swab from a guinea pig, and in the faeces of a sheep. In our study, *A.* *pseudolwoffii* was also most often detected in domestic ruminants, such as cattle, sheep and goats (*n* = 17, prevalence 0.27%), in solipeds such as horses (*n* = 4, prevalence 2.58%), domestic rodents, such as rabbits and guinea pigs (*n* = 2, prevalence 1.18%) and also in domestic carnivores such as dogs and cats (*n* = 2, prevalence 0.09%). Table 2 shows detailed information and a comparison with the occurrence of *A.* *pseudolwoffii* and other *Acinetobacter* species in domestic animals. Literature sources so far mention the isolation of *A.* *baumannii*, *A.* *pittii*, *A.* *calcoaceticus*, *A.* *towneri*, *A.* *johnsonii*, *A.* *lwoffii* and *A.* *radioresistens* from carnivores (Francey et al. 2000; Kuzi et al. 2016; Kimura et al. 2017), horses (Jokisalo et al. 2010; Bzdil et al. 2018) and other animals (Almasaudi 2018). In terms of organs and organ systems, *A.* *pseudolwoffii* was most often isolated from the respiratory tract (*n* = 18, prevalence 3.31%), from the digestive tract (*n* = 4, prevalence 0.28%), from the eye (*n* = 1, prevalence 0.43%) and from the ear and skin (both *n* = 1, prevalence 0.17%). Details of the numbers of *Acinetobacter* spp. isolates and their prevalence in relation to organs and organ systems are provided in Table 3. The specific clinical diagnoses in animals with findings of *A.* *pseudolwoffii* are shown in Table 4. Infections of the respiratory tract and eye caused by *Acinetobacter* spp., both in animals and in humans, are confirmed by literature sources (Francey et al. 2000; Jokisalo et al. 2010; Almasaudi 2018). For example, Almasaudi (2018) mentions wound infections in human patients caused by *Acinetobacter* spp. in his study. Differences in prevalence among individual species and groups of animals as well as among individual organs and organ systems were found in the present study. The varying frequency of findings of individual *Acinetobacter* species could indicate different degrees of affinity to particular animal groups and species, as well as to different organs and organ systems. The variation could be attributed to the specific biochemistry and microclimatic conditions within each organ as well as the diverse geographic, climatic, dietary, and social factors unique to each animal, including humans.

**Table 2:** Number of *Acinetobacter* spp. isolates from individual animal groups and their prevalence (%) in the period from January 1, 2014 to August 31, 2015

Animal	Domestic carnivores	Domestic ruminants	Domestic pigs	Domestic solipeds	Domestic birds	Domestic rodents	Domestic reptiles	Domestic insects (bees)	Total No. of isolates
*Acinetobacter* species									
*A. baumannii*	1 (0.04)	0	0	1 0.65)	0	1 (0.59)	0	0	3 (0.03)
*A. calcoaceticus*	2 0.09)	1 (0.02)	0	1 (0.65)	1 (0.3)	1 (0.59)	1 (2.63)	1 (0.47)	8 (0.08)
*A. gandensis*	0	2 (0.03)	0	0	0	0	0	0	2 (0.02)
*A. guillouiae*	0	1 (0.02)	0	0	0	1 (0.59)	0	0	2 (0.02)
*A. indicus*	0	3 (0.05)	0	0	0	0	0	0	3 (0.03)
*A. johnsonii*	1 (0.04)	2 (0.03)	0	2 (1.29)	3 (0.9)	0	0	0	8 (0.08)
*A. lwoffii*	13 (0.56)	1 (0.02)	0	0	4 (1.2)	1 (0.59)	1 (2.63)	1 (0.47)	21 (0.22)
*A. modestus*	1 (0.04)	0	0	0	0	0	0	0	1 (0.01)
*A. parvus*	1 (0.04)	0	0	0	0	0	0	0	1 (0.01)
*A. pittii*	12 (0.52)	0	0	1 (0.65)	2 (0.6)	3 (1.78)	0	1 (0.47)	19c (0.2)
*A. pseudolwoffii*	2 (0.09)	17 (0.27)	0	4 (2.58)	0	2 (1.18)	0	0	25 (0.26)
*A. radioresistens*	5 (0.22)	0	0	0	0	0	0	0	5 (0.05)
*A. schindleri*	1 (0.04)	1 (0.02)	0	0	0	1 (0.59)	0	0	3 (0.03)
*A. ursingii*	2 (0.09)	0	0	0	0	0	0	0	2 (0.02)
*Acinetobacter* spp. ungrouped	1 (0.04)	0	0	3 (1.94)	0	1 (0.59)	0	0	5 (0.05)
Total No. of isolates	42 (1.81)	28 (0.45)	0	12 (7.74)	10 (3.0)	11 (6.51)	2 5.26)	3 (1.42)	108 (1.13)

**Table 3:** Number of *Acinetobacter* spp. isolates from individual organs and organ systems and their prevalence (%) in the period from January 1, 2014 to August 31, 2015

Organ (system)	Eye	Ear	Skin	Respiratory	Digestive	Urogenital	Milk	Circulation	Total No. of isolates
*Acinetobacter* species									
*A. baumannii*	0	0	1 (0.17)	1 (0.18)	1 (0.07)	0	0	0	3 (0.03)
*A. calcoaceticus*	1 (0.43)	0	3 (0.52)	0	3 (0.21)	1 (0.29)	0	0	8 (0.08)
*A. gandensis*	1 (0.43)	0	0	0	0	0	1 (0.02)	0	2 (0.02)
*A. guillouiae*	0	0	0	1 (0.18)	0	0	1 (0.02)	0	2 (0.02)
*A. indicus*	0	0	0	0	1 (0.07)	0	2 (0.03)	0	3 (0.03)
*A. johnsonii*	0	0	1 (0.17)	3 0.55)	3 (0.21)	0	1 (0.02)	0	8 (0.08)
*A. lwoffii*	4 (1.71)	1 (0.17)	6 (1.04)	2 (0.37)	8 (0.56)	1 (0.29)	1 (0.02)	1 (4.55)	24 (0.25)
*A. modestus*	0	0	0	1 (0.18)	0	0	0	0	1 (0.01)
*A. parvus*	0	0	0	1 (0.18)	0	0	0	0	1 (0.01)
*A. pittii*	1 (0.43)	2 (0.33)	9 (1.56)	2 (0.37)	2 (0.14)	3 (0.87)	0	0	19 (0.2)
*A. pseudolwoffii*	1 (0.43)	1 (0.17)	1 (0.17)	18 (3.31)	4 (0.28)	0	0	0	25 (0.26)
*A. radioresistens*	0	0	3 (0.52)	0	1 (0.07)	1 (0.29)	0	0	5 (0.05)
*A. schindleri*	0	0	1 (0.17)	0	2 (0.14)	0	0	0	3 (0.03)
*A. ursingii*	0	0	1 (0.17)	1 (0.18)	0	0	0	0	2 (0.02)
*Acinetobacter* sp. (ungrouped)	0	0	0	0	1 (0.07)	1 (0.29)	0	0	2 (0.02)
Total No. of isolates	8 (3.42)	4 (0.67)	26 (4.5)	30 (5.51)	26 (1.83)	7 (2.03)	6 (0.1)	1 (4.55)	108 (1.13)

**Table 4:** Findings of *Acinetobacter pseudolwoffii* in individual groups, species and categories of animals, diagnoses and occurrence of multi-drug resistant strains

Group of animals	Number of samples	Species or category of animals	Number of samples	Diagnosis	Number of cases	Multi-drugresistant strains	Number of isolates
Ruminants	17	calf	11	Bronchitis acuta	2	–	–
				Bronchitis purulenta acuta	1	DO, SXT, CAZ	1
				Bronchopneumonia acuta	5	–	–
				Bronchopneumoniaet myocarditis chronica	1	–	–
				Conjunctivitis acuta	1	DO, SXT, CAZ	1
				Rhinitis purulenta acuta	1	–	–
		cow	4	Bronchopneumonia acuta	2	COT, PRL, CAZ	1
				Bronchopneumonia chronica	1	–	–
				Dermatitis interdigitalis	1	–	–
		sheep	1	Enteritis acuta	1	–	–
		goat	1	Rhinitis purulenta chronica	1	–	–
							
Solipeds	4	horse	4	Bronchopneumoniaet rhinitis acuta	2	OFX, SAM, CN, SXT, PRL, CAZ	1
				Nasopharyngitis acuta	1	–	–
				Enteritis acuta	1	–	–
							
Rodents	2	rabbit	1	Enteritis acuta	1	–	–
		guinea pig	1	Enteritis acuta	1	–	–
							
Carnivores	2	dog	1	Otitis externa acuta	1	–	–
		cat	1	Rhinitis purulenta chronica	1	–	–

Multi-drug resistant strains = resistance to 3 and more antimicrobials

CAZ = ceftazidime; CN = gentamicin; DO = doxycycline; OFX = ofloxacin; PRL = piperacillin; SAM = ampicillin/sulbactam; SXT = co-trimoxazole

Further studies in different countries and regions worldwide are needed to confirm or refute this assumption. Antimicrobial susceptibility tests were performed on all 25 of our *A.* *pseudolwoffii* isolates and 83 other detected *Acinetobacter* spp. isolates. *A.* *pseudolwoffii* was susceptible in all cases to imipenem, meropenem, tobramycin, amikacin and netilmicin. For ceftazidime, only 32% of the isolates demonstrated susceptibility, while 60% were susceptible to co-trimoxazole, 80% to piperacillin, 88% to doxycycline, and 96% to ampicillin/sulbactam, gentamicin, and ofloxacin. Notably, one of the tested *A.* *lwoffii* isolates out of 21 tested isolates, was resistant to imipenem and meropenem (susceptibility 95.2%). Table 5 shows the percentage of susceptible bacterial isolates out of the total number tested. The highest resistance to ceftazidim is not surprising. The *AmpC Acinetobacter*-derived cephalosporinase encoded by the *bla*_ADC_ gene is responsible for resistance to ceftazidime, which is described in isolates of *Acinetobacter* spp. obtained from humans. This gene has been described in up to 99% of strains (Hujer et al. 2006). Therefore, its presence can also be assumed in strains in the animal population.

**Table 5:** Susceptibility of *Acinetobacter* spp. isolates from animals to antimicrobials – susceptible/examined isolates (% susceptible) in the period from January 1, 2014 to August 31, 2015

Antimicrobials	Imipenem	Meropenem	Ampicillin/Sulbactam	Piperacillin	Ceftazidim	Gentamicin	Tobramycin	Amikacin	Netilmicin	Ofloxacin	Doxycycline	Co-trimoxazole
Strain												
*A. baumannii*	3/3 (100)	3/3 (100)	2/3 (66.7)	2/3 (66.7)	1/3 (33.3)	2/3 (66.7)	3/3 (100)	3/3 (100)	2/3 (66.7)	2/3 (66.7)	2/3 (66.7)	2/3 (66.7)
*A. calcoaceticus*	8/8 (100)	8/8 (100)	8/8 (100)	6/8 (75.0)	3/8 (37.5)	8/8 (100)	8/8 (100)	8/8 (100)	8/8 (100)	8/8 (100)	8/8 (100)	8/8 (100)
*A. gandensis*	2/2 (100)	2/2 (100)	2/2 (100)	1/2 (50)	0/2 (0)	2/2 (100)	1/2 (50)	1/2 (50)	2/2 (100)	1/2 (50)	2/2 (100)	1/2 (50)
*A. guillouiae*	2/2 (100)	2/2 (100)	2/2 (100)	2/2 (100)	2/2 (100)	2/2 (100)	2/2 (100)	2/2 (100)	2/2 (100)	2/2 (100)	2/2 (100)	2/2 (100)
*A. indicus*	3/3 (100)	3/3 (100)	3/3 (100)	3/3 (100)	3/3 (100)	3/3 (100)	3/3 (100)	3/3 (100)	3/3 (100)	3/3 (100)	3/3 (100)	3/3 (100)
*A. johnsonii*	8/8 (100)	8/8 (100)	8/8 (100)	8/8 (100)	5/8 (62.5)	7/8 (87.5)	8/8 (100)	8/8 (100)	8/8 (100)	7/8 (87.5)	8/8 (100)	7/8 (87.5)
*A. lwoffii*	20/21 (95.2)	20/21 (95.2)	21/21 (100)	18/21 (85.7)	17/21 (81.0)	20/21 (95.2)	20/21 (95.2)	21/21 (100)	20/21 (95.2)	19/21 (90.5)	21/21 (100)	18/21 (85.7)
*A. modestus*	1/1 (100)	1/1 (100)	1/1 (100)	1/1 (100)	1/1 (100)	1/1 (100)	1/1 (100)	1/1 (100)	1/1 (100)	1/1 (100)	1/1 (100)	1/1 (100)
*A. parvus*	1/1 (100)	1/1 (100)	1/1 (100)	1/1 (100)	1/1 (100)	1/1 (100)	1/1 (100)	1/1 (100)	1/1 (100)	1/1 (100)	1/1 (100)	1/1 (100)
*A. pittii*	19/19 (100)	19/19 (100)	19/19 (100)	17/19 (89.5)	17/19 (89.5)	19/19 (100)	19/19 (100)	19/19 (100)	18/19 (94.7)	19/19 (100)	19/19 (100)	18/19 (94.7)
*A. pseudolwoffii*	25/25 (100)	25/25 (100)	24/25 (96.0)	20/25 (80.0)	8/25 (32.0)	24/25 (96.0)	25/25 (100)	25/25 (100)	25/25 (100)	24/25 (96.0)	22/25 (88.0)	15/25 (60.0)
*A. radioresistens*	5/5 (100)	5/5 (100)	5/5 (100)	4/5 (80.0)	5/5 (100)	5/5 (100)	5/5 (100)	5/5 (100)	5/5 (100)	5/5 (100)	5/5 (100)	5/5 (100)
*A. schindleri*	3/3 (100)	3/3 (100)	3/3 (100)	3/3 (100)	3/3 (100)	3/3 (100)	3/3 (100)	3/3 (100)	3/3 (100)	3/3 (100)	3/3 (100)	3/3 (100)
*A. ursingii*	2/2 (100)	2/2 (100)	2/2 (100)	2/2 (100)	1/2 (50)	2/2 (100)	2/2 (100)	2/2 (100)	2/2 (100)	2/2 (100)	2/2 (100)	2/2 (100)
*Acinetobacter* spp. (ungrouped)	5/5 (100)	5/5 (100)	5/5 (100)	5/5 (100)	3/5 (60.0)	5/5 (100)	5/5 (100)	5/5 (100)	5/5 (100)	5/5 (100)	5/5 (100)	3/5 (60.0)
Total susceptible	107/108 (99.1)	107/108 (99.1)	106/108 (98.1)	93/108 (86.1)	70/108 (64.8)	104/108 (96.3)	106/108 (98.1)	107/108 (99.1)	105/108 (97.2)	102/108 (94.4)	104/108 (96.3)	89/108 (82.4)

The above-mentioned Table 4 also shows the occurrence of multi-drug resistant isolates (MDR) of *A. pseudolwoffii* in different animal groups and species. The two isolates of *A. pseudolwoffii* found in calves were simultaneously resistant to doxycycline, co-trimoxazole, and ceftazidime and one isolate from an adult cow was resistant to co-trimoxazole, piperacillin and ceftazidime. Although there is no existing literature on the multi-drug resistance of *A.* *pseudolwoffii*, its resistance is relatively low when compared to *Acinetobacter* spp. in some previous studies. For example, in the USA a total of 54% of *Acinetobacter* spp. strains isolated from human patients were MDR (Queenan et al. 2012). One strain isolated from a horse showed resistance to ofloxacin, gentamicin, ampicillin/sulbactam, co-trimoxazole, piperacillin, and ceftazidime.

However, Bzdil et al. (2018) reported different findings for strains isolated from horses, indicating 100% sensitivity to gentamicin, colistin and co-trimoxazole. Sensitivities to neomycin, tetracyclines and fluoroquinolones ranged between 90 and 95.2%, while sensitivities to florfenicol, streptomycin and amoxicillin with clavulanic acid ranged between 71.4 and 83.3%. For cephalothin, lincosamides and macrolides sensitivities ranged between 5.9 and 35%. Jokisalo et al. (2010) detected susceptibility only to fluoroquinolones and co-trimoxazole in a multiresistant strain of *A.* *baumannii* isolated from horses. High antimicrobial resistance was confirmed by molecular genotyping methods in 22 strains of *A.* *baumannii* isolated from meat by Tavakol et al. (2018). They demonstrated resistance genes to tetracycline in 90.9% of strains, to co-trimoxazole in 54.5% of strains, and to gentamicin in 50% of strains. The increase of resistance to carbapenems was confirmed, for example, by Gentilini et al. (2018) in the species *A.* *baumannii* and *A.* *radioresistens*.

The present study describes the occurrence of *A.* *pseudolwoffi* in the context of other species of *Acinetobacter* spp. concurrently identified in clinical samples collected from domestic animals in the Czech Republic. It assesses their susceptibility to antimicrobials, examines the occurrence of multi-drug resistance in the isolates, and also presents the clinical diagnoses of animals with *A.* *pseudolwoffii*. Some of the *A.* *pseudolwoffii* isolates were included in a descriptive taxonomic study in 2019 (Nemec et al. 2019).

The findings of the present study hold the potential to benefit both the scientific community and clinical practice.
